# The Flavonoid Rutin Enhances Temozolomide Sensitivity in Glioblastoma Spheroids by Modulating Chemoresistance via PI3K/AKT, STAT3, Redox and Kynurenine Pathways, and Altering ECM Remodeling Associated with Reduced Migration

**DOI:** 10.3390/antiox15050643

**Published:** 2026-05-19

**Authors:** Irlã Santos Lima, Fernanda Vidal Carvalho, Érica Novaes Soares, Monique Reis de Santana, Maria de Fátima Dias Costa, Carolina Kymie Vasques Nonaka, Bruno Solano de Freitas Souza, Henning Ulrich, Cleonice Creusa dos Santos, Silvia Lima Costa

**Affiliations:** 1 Laboratory of Neurochemistry and Cellular Biology (LabNq), Institute of Health Sciences, Federal University of Bahia, Salvador 40231-300, BA, Brazil; sci.lima@outlook.com (I.S.L.); vidal.fernanda13@gmail.com (F.V.C.); ericanovaessoares@gmail.com (É.N.S.); moniquereisant@gmail.com (M.R.d.S.); fatima@ufba.br (M.d.F.D.C.); 2 Center of Biotechnology and Cell Therapy (CBCT), São Rafael Hospital, D’Or Institute for Research and Teaching, Salvador 41253-190, BA, Brazil; carolina.nonaka@hsr.com.br (C.K.V.N.); bruno.solano@idor.org.br (B.S.d.F.S.); 3 Department of Biochemistry, Institute of Chemistry, University of São Paulo, São Paulo 05508-900, SP, Brazil; henning@iq.usp.br; 4 National Institute of Translational Neuroscience (INNT), Rio de Janeiro 21941-902, RJ, Brazil

**Keywords:** glioblastoma, rutin, oxidative stress, kynurenine metabolism, temozolomide, microtumors, PI3K/AKT, chemoresistance, extracellular matrix remodeling, STAT3

## Abstract

Introduction: Glioblastoma (GBM) is the most aggressive primary tumor of the central nervous system and is highly resistant to temozolomide (TMZ). Rutin is a potent antioxidant with immunomodulatory and anti-glioma effects in vitro, although its mechanisms of action remain incompletely understood. This study investigated the effects of rutin on morphology, viability, redox balance, and pro-tumoral signaling in GBM 2D cultures and 3D spheroids, as well as its association with TMZ sensitivity. Methods: GL15 and U343 human GBM cell lines and primary astrocytes were treated with rutin (5–30 μM) and/or TMZ (125–4000 μM). Cell metabolic activity and viability were assessed by MTT, PI/DiOC18(3) or PI/Hoechst. Cell migration was assessed from spheroid-derived cells, and extracellular matrix (ECM) components (fibronectin and laminin) were evaluated by immunofluorescence. Intracellular reactive oxygen species (ROS) were measured by DCFH-DA fluorescence. IL-6, STAT3, NOS2, and IDO1 gene expression were determined by RT-qPCR, and protein expression of MMP2, fibronectin, STAT3, PI3K, and AKT by Western blotting. Nitric oxide (NO) and L-kynurenine levels were quantified in the supernatant by colorimetric assays. Results: Rutin reduced cell viability and enhanced TMZ cytotoxicity in both 2D and 3D cultures, while exerting selective effects by increasing metabolic activity and attenuating TMZ-induced effects in non-tumoral primary astrocytes. In 3D spheroids, rutin affected structural organization and reduced spheroid-derived cell migration, accompanied by changes in ECM components, including MMP2, fibronectin, and laminin. Rutin decreased intracellular ROS levels and suppressed the TMZ-induced increase in ROS and NOS signaling. These effects were accompanied by modulation of IL-6/STAT3 signaling, along with reduced STAT3, PI3K, and AKT protein levels. Rutin also modulated immunometabolic parameters, including extracellular L-kynurenine and nitric oxide levels, and enhanced TMZ responsiveness following pre-sensitization. Conclusions: Rutin enhances TMZ responsiveness by modulating interconnected pro-tumoral mechanisms, including redox balance, pro-survival signaling, ECM remodeling and migratory behavior, and immunometabolic pathways linked to chemoresistance, supporting its potential as an adjuvant therapeutic strategy.

## 1. Introduction

Glioblastoma (GBM) is the most aggressive primary brain tumor in adults and is defined as a grade IV astrocytoma, IDH-wildtype, according to the World Health Organization classification, characterized by rapid growth, diffuse infiltration, and poor prognosis [1]. Despite the current standard of care, including maximal surgical resection followed by radiotherapy with concomitant and adjuvant temozolomide (TMZ), patient survival remains limited, with a median overall survival of approximately 15 months [2].

Accumulating evidence indicates that resistance to TMZ is not solely driven by genetic alterations but also by adaptive responses within the tumor microenvironment. In particular, TMZ exposure promotes intracellular accumulation of reactive oxygen species (ROS), disrupting redox homeostasis and triggering compensatory survival mechanisms [3,4]. This redox imbalance is closely linked to the activation of pro-survival signaling pathways, including PI3K/AKT and JAK/STAT3, often sustained by inflammatory mediators such as IL-6, thereby promoting tumor cell survival, proliferation, and resistance to therapy [5]. In parallel, redox and inflammatory signaling are tightly interconnected with tumor immunometabolism. Activation of the indoleamine-2,3-dioxygenase (IDO) pathway promotes tryptophan catabolism and kynurenine production, which not only drive immune evasion but also modulate cellular redox balance and metabolic adaptation [6,7]. Additionally, induction of NOS2 and increased nitric oxide (NO) production further modulate redox signaling and tumor progression, reinforcing pro-tumoral signaling within the tumor microenvironment [8]. These molecular adaptations are closely associated with structural remodeling processes within the tumor microenvironment. Extracellular matrix (ECM) components, such as fibronectin and laminin, together with matrix metalloproteinases (e.g., MMP2), regulate cell adhesion, migration, and invasion [9]. Beyond facilitating tumor dissemination, ECM remodeling functionally interacts with intracellular signaling networks, modulating pro-survival pathways associated with tumor progression and chemoresistance [10].

Given the complexity of interactions in the GBM microenvironment, experimental models that partially recapitulate key features of the tumor are essential for studying GBM biology. While two-dimensional (2D) cultures are widely used, they fail to mimic the complex three-dimensional (3D) architecture and cell–matrix interactions found in vivo. In contrast, 3D spheroids better recapitulate aspects of tumor heterogeneity, metabolic gradients, and diffusion limitations, contributing to increased resistance to chemotherapeutic agents and providing a more physiologically relevant model [11]. Also, increasing attention has been directed toward adjuvant and combination therapeutic strategies to improve GBM treatment outcomes, particularly approaches integrating chemotherapeutic agents with compounds capable of modulating oxidative stress, inflammatory signaling, and cell death pathways [12].

In this framework, combination strategies integrating natural compounds with chemotherapeutic agents have emerged as a promising approach in GBM treatment. In particular, phytotherapy-based approaches have gained attention due to their anti-tumor, anti-inflammatory, and immunomodulatory properties, supporting their investigation as adjuvant therapeutic strategies [13]. Rutin (quercetin-3-O-rutinoside), a flavonoid with well-established antioxidant, immunomodulatory, and neuroprotective properties, has been shown to modulate oxidative stress, intracellular signaling pathways, and tumor cell viability [14,15,16]. Previous studies in glioma models indicate that rutin can interfere with processes such as proliferation, apoptosis, autophagy, and microRNA regulation [17,18]. However, these studies have primarily focused on isolated mechanisms and were largely conducted in two-dimensional systems. Its impact on integrated mechanisms of chemoresistance, particularly in the context of redox balance, inflammatory signaling, immunometabolism, and ECM remodeling, remains poorly understood, especially in 3D spheroid models. Based on this rationale, the present study investigated whether rutin modulates the redox–inflammatory–immunometabolic axis and ECM dynamics associated with TMZ resistance in GBM. Using 3D spheroid models that better recapitulate key features of spheroid architecture, including cell–cell interactions and diffusion gradients, we evaluated the effects of rutin, alone and in combination with TMZ, on spheroid morphology, viability, migration, oxidative stress, and key signaling pathways involved in GBM progression and chemoresistance.

## 2. Materials and Methods

### 2.1. Cell Line Culture

The GL15 cell line was established and initially characterized from a human GBM (hGBM) by Bocchini et al. (1991) [19], with further molecular characterization reported in subsequent studies [20]. The U343 cell line, derived from human high-grade astrocytomas (grade III–IV), was established by Westermark et al. (1973) [21] and further characterized by Bigner et al. (1981) [22]. These were used in this study (passages 20–50). Both cell lines were used as hGBM models due to their high-grade glioma origin and widely reported tumorigenic properties. These hGBM cell lines were selected due to their distinct proliferation, migration, and chemoresistance profiles, as well as differences in spheroid architecture and morphological organization. Cells were cultured in Dulbecco’s Modified Eagle Medium (DMEM; Gibco; Grand Island, NY, USA) containing 7 mmol/L glucose, 2 mmol/L L-glutamine, and 0.011 g/L pyruvic acid, as previously described by Lima et al. (2024) [18], supplemented with 10% fetal bovine serum (FBS) and 1% penicillin–streptomycin (Gibco; Thermo Fisher Scientific; Waltham, MA, USA). Cultures were maintained at 37 °C in a humidified atmosphere of 5% CO_2_. Cells were washed with sterile phosphate-buffered saline (PBS) and dissociated using trypsin–EDTA solution (0.05% trypsin/0.02% EDTA in PBS) for experimental procedures. Enzymatic activity was neutralized with FBS-supplemented DMEM, and cells were centrifuged at 2000 rpm for 5 min, resuspended, and counted using a hemocytometer. For two-dimensional (2D) assays, cells were maintained in DMEM supplemented with 10% FBS. For three-dimensional (3D) assays, including spheroid formation, cells were cultured in DMEM without FBS.

### 2.2. Primary Cortical Astrocyte Cultures

Primary astrocytes were obtained from neonatal C57BL/6 mice (postnatal days 0–2), provided by the Animal Facility of the Institute of Health Sciences (ICS), Federal University of Bahia (UFBA), Salvador, Brazil. All procedures were conducted at the Laboratory of Neurochemistry and Cell Biology (LabNq), ICS-UFBA, in accordance with the local Ethics Committee for Animal Experimentation (CEUA; protocol No. 2485270223, approved on 16 June 2023). Following decapitation, cerebral hemispheres were aseptically dissected, and meninges and blood vessels were carefully removed. Cortical tissue was mechanically dissociated by gentle trituration in DMEM containing 7 mmol/L glucose, 2 mmol/L L-glutamine, and 0.011 g/L pyruvic acid, supplemented with 10% FBS and 1% penicillin–streptomycin. The resulting cell suspension was transferred to poly-D-lysine-coated (0.1 mg/mL; Sigma-Aldrich; St. Louis, MO, USA) 75 cm^2^ culture flasks (Corning, NY, USA). Cells were maintained in DMEM supplemented with 10% FBS and 1% penicillin–streptomycin at 37 °C in a humidified atmosphere of 5% CO_2_, with medium replacement every 2–3 days. After 7 days in culture, cells were subjected to orbital shaking (180 rpm, 37 °C) for 3 h to remove loosely attached microglial cells. The supernatant was discarded, and astrocytes were dissociated using trypsin–EDTA solution (0.05% trypsin/0.02% EDTA in PBS) for 5 min at 37 °C. Enzymatic activity was neutralized with FBS-supplemented DMEM, and the cell suspension was collected, centrifuged at 1000 rpm for 10 min, and resuspended in fresh supplemented medium. Cells were counted using a hemocytometer and prepared for subsequent viability assays.

### 2.3. Preparation and Storage of Compounds

To evaluate chemotherapy-induced stress responses and their modulation in hGBM and primary mouse astrocyte cells, as well as to provide a non-tumoral glial reference for comparison, temozolomide (TMZ; T2577; Sigma-Aldrich; St. Louis, MO, USA), the standard chemotherapeutic agent [23], and rutin (quercetin-3-O-rutinoside; R5143; Merck; Boston, MA, USA), a bioactive flavonoid with reported antioxidant, anti-inflammatory, and antitumoral effects in glioma models, were employed [24]. TMZ was dissolved in dimethyl sulfoxide (DMSO; Sigma-Aldrich; St. Louis, MO, USA) to obtain a 50 mM stock solution, which was aliquoted and stored at −20 °C protected from light, with each aliquot thawed only once prior to use. It was applied at concentrations ranging from 125 to 4000 µM, based on previous studies demonstrating dose-dependent cytotoxic and adaptive responses in glioma models [25]. Similarly, rutin was dissolved in DMSO to prepare a 100 mM stock solution and stored at 4–8 °C under light-protected conditions. It was used at concentrations ranging from 5 to 30 µM, consistent with a previously reported IC_50_ of approximately 30 µM in GBM-related models [18]. For all experiments, both compounds were freshly diluted in serum-free DMEM immediately before use, ensuring that the final DMSO concentration did not exceed 1% (*v*/*v*). Sub-IC_50_ conditions were selected to allow the assessment of treatment-associated molecular responses while limiting the contribution of extensive cytotoxicity.

### 2.4. Cell Viability Assay in 2D Monolayer Cultures

To assess cell viability and metabolic activity, the 3-(4,5-dimethylthiazol-2-yl)-2,5-diphenyltetrazolium bromide (MTT; Invitrogen; Thermo Fisher Scientific; Waltham, MA, USA) reduction assay was used to evaluate the effects of rutin alone and in combination with TMZ on hGBM cells in 2D monolayer cultures. This assay monitors metabolic activity and cell viability over a short time period. To determine the selectivity of the compounds toward tumor cells, we also evaluated their effects on cortical astrocytes as a non-tumoral control. For this, GL15, U343 and mouse cortical astrocytes were seeded in 96-well plates (Kasvi; São José dos Pinhais, SP, Brazil) at densities optimized for each cell type. hGBM cells were plated at 2.5 × 104 cells/cm^2^ (approximately 8.0 × 103 cells per well) and maintained for 24 h prior to treatment. Astrocytes were seeded at 5.0 × 104 cells/cm^2^ (approximately 1.6 × 104 cells per well) and maintained for 5 days prior to treatment, with the medium replaced every 2–3 days to allow stabilization. All cultures were maintained in DMEM supplemented with 10% FBS under a humidified atmosphere of 5% CO_2_ at 37 °C. After the adherence period, the cultures were exposed for 48 h to TMZ (125–4000 µM), rutin (5–30 µM), or a combination of both, while the controls received only DMSO at equivalent concentrations. At the end of the treatments, MTT solution (0.5 mg/mL in DMEM) was added and the plates incubated for 2 h at 37 °C. The medium was then removed, and 100 µL of DMSO was added to solubilize the formazan crystals, followed by incubation for 10–15 min under gentle agitation. Absorbance was measured at 540 nm using a microplate reader (Varioskan™ LUX multimode microplate reader, Thermo Fisher Scientific; Waltham, MA, USA). Three independent experiments were performed with eight technical replicates per condition, and results were expressed as a percentage relative to the control.

### 2.5. Formation of 3D hGBM Spheroids

For 3D culture, hGBM cells were arranged in agarose micromolds (12–81; MicroTissues 3D Petri Dish^®^; Millipore Sigma; St. Louis, MO, USA). The molds were prepared with 2% (*w*/*v*) agarose (Agargen; Cat. 6108; Madrid, Spain) in 0.9% NaCl solution, poured while still hot into the wells and allowed to solidify for 2 to 5 min. The solidified micromolds were then transferred to 12-well plates (Kasvi; São José dos Pinhais, SP, Brazil) and stabilized with a thin layer of agarose. After three washes with serum-free DMEM for equilibration, each micromold received approximately 3375 cells per microwell, totaling about 273,000 cells per mold. After seeding, the micromolds containing the cells were incubated for 60 min at 37 °C and 5% CO_2_ to allow cell sedimentation and the beginning of the formation of 3D aggregates. Then, 2 mL of serum-free DMEM was added per well. The spheroids were cultured for periods between 4 and 9 days, according to the experimental design, with medium changes every 48 h. Development and morphology were monitored daily by phase-contrast microscopy.

To quantitatively assess spheroid structural organization and morphological changes under different experimental conditions, radiomic analysis was performed using AnaSP 3.0 software (University of Bologna, Bologna, Italy), according to the developer documentation and previous validation studies [26]. Spheroids were manually segmented, and morphometric features were extracted, including equivalent diameter, circularity, compactness, convexity, solidity, sphericity, and entropy. Equivalent diameter (√(4A/pi)) was used as a normalized measure of spheroid size, reflecting overall growth independent of shape irregularities. Circularity and compactness were calculated as 4piA/(ConvexPerimeter)2 and 4piA/(Perimeter)2, respectively, representing shape regularity and structural cohesion. Convexity (ConvexPerimeter/Perimeter) was defined as the ratio between convex and actual perimeter, indicating boundary smoothness and edge irregularity. Solidity (Area/ConvexArea) reflects the degree of compactness relative to a convex shape, while sphericity (pi√(4A/pi)/Perimeter) describes three-dimensional organization and structural symmetry. Entropy (∑pilog2(pi)), calculated from the normalized gray-level histogram, was used to assess structural heterogeneity and internal disorder, as implemented in the AnaSP software. Collectively, these parameters reflect biologically relevant features of spheroid organization, including structural cohesion, integrity, and internal heterogeneity, which are associated with treatment response in 3D tumor models [27]. Together, these parameters provide an integrated assessment of spheroid size, morphology, and structural organization, enabling comparison between experimental conditions. For spheroid-based analyses, eight individual spheroids per condition were analyzed per experiment, and the mean value was used as a single data point for each of three independent biological replicates.

### 2.6. Spheroid-Derived Cell Migration Analysis

To evaluate the migratory potential of hGBM cells derived from pre-established spheroids, a migration assay was performed using spheroids cultured for 7 days to allow structural maturation. Individual spheroids were carefully transferred to wells of adherent 96-well plates containing the respective treatments: DMSO, TMZ (500–1000 µM), rutin (5–15 µM), or TMZ (500 µM) combined with rutin (5–15 µM). Cultures were maintained under standard incubation conditions and monitored daily by phase-contrast microscopy. Images were acquired to assess spheroid-derived cell migration. Migration was quantified by measuring the distance from the spheroid edge to the furthest migrating cell, defined as the distance between the spheroid edge and the most distant migrating cell. In addition, the number of migrating cells was estimated, and the presence of fibronectin and laminin was evaluated after 72 h of culture. All experiments were performed in biological triplicates.

### 2.7. Immunofluorescence Detection of Fibronectin and Laminin

To evaluate the presence and distribution of proteins associated with cell adhesion during spheroid-derived cell migration, immunofluorescence staining for fibronectin and laminin was performed using cultures obtained from the spheroid migration assay. After 72 h of treatment, cultures were fixed with cold methanol for 10 min at −20 °C. Samples were washed three times with PBS and blocked with 5% bovine serum albumin (BSA; Sigma-Aldrich; St. Louis, MO, USA) in PBS for 1 h at room temperature. Cells were then incubated with primary antibodies diluted in PBS containing 1% BSA under gentle agitation for 3 h in the dark. The following primary antibodies were used: anti-fibronectin (rabbit polyclonal; 1:40; F3648; Sigma-Aldrich; St. Louis, MO, USA; RRID: AB_476976) and anti-laminin (LAMA1) (rabbit polyclonal; 1:200; L9393; Sigma-Aldrich; St. Louis, MO, USA; RRID: AB_477163). After incubation, cultures were washed three times with PBS and incubated with Alexa Fluor 594-conjugated anti-rabbit IgG (H + L) secondary antibody (1:1000) for 1 h at room temperature under gentle agitation and protected from light. Cells were subsequently washed three times with PBS and counterstained with 5 μg/mL of 4′,6-diamidino-2-phenylindole (DAPI; Molecular Probes; Eugene, OR, USA) for 5 min. Fluorescent images were acquired using a fluorescence microscope (Leica DFC7000; Wetzlar, Germany), and fluorescence intensity was quantified using FIJI/ImageJ 1.54p software (National Institutes of Health; Bethesda, MD, USA). All fluorescence images were acquired using identical exposure and acquisition settings across experimental groups to allow reliable comparison of fluorescence intensity. All experiments were performed in biological triplicates.

### 2.8. Fluorescent Staining for Spheroid Viability Assessment

Cell viability and cellular density in hGBM spheroids were evaluated using fluorescent markers in independent experiments. Depending on the experimental design, spheroids were labeled either with 5 µg/mL of Hoechst 33342 (Cat. 33342; Thermo Fisher Scientific; Waltham, MA, USA) for nuclear staining or with 20 µg/mL of the lipophilic dye DiOC18(3) (3,3′-dioctadecyloxacarbocyanine perchlorate; Cat. D275; Invitrogen; Life Technologies; Carlsbad, CA, USA) which is incorporated in intact cellular membranes and is commonly used for cell tracking and viability analysis in 2D and 3D culture systems. The DiOC18(3) stock solution (1 mg/mL) was prepared in sterile DMSO and homogenized in an ultrasonic bath at 50 °C for 30 min to minimize dye aggregation. For experiments using DiOC18(3), monolayer cultures were incubated with the dye diluted in serum-free medium for 15 min at 37 °C on the day prior to spheroid formation. Cells were then washed three times with sterile PBS to remove excess dye and maintained in complete medium for 24 h before seeding into micromolds. For the detection of nonviable cells, propidium iodide (PI; Thermo Fisher Scientific; Waltham, MA, USA) was used as a nuclear marker of membrane-compromised cells. Fluorescent images were acquired using a fluorescence microscope (Leica DFC7000; Wetzlar, Germany), and fluorescence intensity was quantified using FIJI/ImageJ 1.54p software (National Institutes of Health; Bethesda, MD, USA). All fluorescence images were acquired using identical exposure and acquisition settings across experimental groups to allow reliable comparison of fluorescence intensity. All experiments were performed in biological triplicates.

### 2.9. Evaluation of Chemosensitivity in GBM Cell Cultures

To assess chemosensitivity, hGBM cells GL15 and U343 were evaluated in both 2D monolayer and 3D spheroid culture models under four conditions: DMSO (0.5%), rutin (5 µM), TMZ (500 µM), and rutin + TMZ. For 2D cultures, cells were seeded and allowed to adhere for 24 h. Subsequently, cells were exposed to rutin, DMSO, or fresh medium. After 24 h, TMZ was added to the appropriate groups, and cultures were maintained for an additional 24 h. Supernatants were collected for L-kynurenine and nitric oxide (NO) quantification, while the corresponding cellular material was harvested for NOS2 expression analysis by RT-qPCR. For spheroid assays, cells were seeded directly into spheroid-forming micromolds and exposed to rutin, DMSO, or fresh medium at the time of plating. After 24 h, TMZ was added to the appropriate groups. Spheroids were maintained for an additional 48 h prior to analysis. Morphological aspects were evaluated using AnaSP software. Cell viability was assessed by PI staining for dead cells and Hoechst staining for total nuclei. Fluorescent images were acquired using a Leica DFC7000 fluorescence microscope, and fluorescence intensity was quantified using FIJI/ImageJ 1.54p software (Wayne Rasband; National Institutes of Health, USA). All experiments were performed in biological triplicates.

### 2.10. Nitric Oxide (NO) Production Assay (Griess Reaction)

To evaluate the involvement of NO in GBM biology and treatment response, NO production was assessed. Elevated NO levels have been associated with increased tumor cell proliferation, survival, and clonogenic potential in GBM, whereas inhibition of nitric oxide synthase (NOS) reduces these tumor-promoting effects [28]. NO production was indirectly assessed by quantifying nitrite levels in culture supernatants using the Griess colorimetric assay. Supernatants were collected from hGBM cultures during spheroid formation and from chemosensitivity experiments. Equal volumes (50 μL) of culture supernatant and Griess reagent (prepared by mixing 1% sulfanilamide with 0.1% N-(1-naphthyl) ethylenediamine dihydrochloride in 2.5% phosphoric acid) were added to 96-well plates and incubated for 10–15 min at room temperature to allow color development. Nitrite concentrations were determined by comparison with a sodium nitrite (NaNO_2_) standard curve ranging from 6.25 to 100 µM NaNO_2_, corresponding to the linear range of the assay, prepared in culture medium. Absorbance was measured at 490 nm using a microplate reader (Varioskan™ LUX multimode microplate reader, Thermo Fisher Scientific; Waltham, MA, USA). All measurements were performed in biological triplicates.

### 2.11. Assessment of L-Kynurenine Levels

To investigate tumor-associated immunometabolic alterations, kynurenine levels were quantified as a readout of tryptophan catabolism through the IDO-dependent kynurenine pathway (KP). Activation of this pathway plays a central role in tumor immune escape and progression, supporting its relevance as a functional marker in GBM [7]. IDO enzymatic activity was indirectly assessed by measuring kynurenine levels in culture supernatants as described [29]. Supernatants were collected from GBM GL15 and U343 cultures during spheroid formation and from chemosensitivity assays. Aliquots of 160 μL of culture supernatant were mixed with 10 μL of 30% trichloroacetic acid (TCA) and incubated at 50 °C for 30 min to hydrolyze N-formylkynurenine into kynurenine. Samples were then centrifuged for 10 min at 600× *g*, and 100 μL of the supernatant were transferred to flat-bottom 96-well plates. Subsequently, 100 μL of Ehrlich’s reagent (1.2% (*w*/*v*) 4-dimethylaminobenzaldehyde in glacial acetic acid) was added to each well. Plates were incubated for 10 min at room temperature, and optical density was measured at 492 nm using a microplate reader (Varioskan™ LUX multimode microplate reader; Thermo Fisher Scientific; Waltham, MA, USA). Kynurenine concentrations were determined by comparison with a standard curve prepared with L-kynurenine sulfate (Sigma-Aldrich; St. Louis, MO, USA). All measurements were performed in biological triplicates.

### 2.12. Gene Expression Analysis by RT-qPCR

To evaluate gene expression in hGBM cells, total RNA was extracted from both stable spheroids and cells obtained from the chemosensitivity assay. For spheroid samples, cultures were washed once with sterile PBS and subjected to two consecutive centrifugations (2000 rpm, 5 min, 4 °C) to obtain a pellet free of culture medium. For the chemosensitivity assay, adherent cultures were washed three times with PBS at the end of treatment. RNA extraction was performed using TRIzol^®^ reagent (Invitrogen; Life Technologies; Carlsbad, CA, USA; 15596026) according to the manufacturer’s instructions. Spheroids were mechanically dissociated using a sterile insulin syringe to ensure complete homogenization. All samples were processed in biological triplicates. RNA concentration and purity were determined using a NanoDrop™ 2000 spectrophotometer (Thermo Fisher Scientific; Waltham, MA, USA), and samples were stored at −80 °C until cDNA synthesis. For reverse transcription, 1.5 µg of total RNA was converted into cDNA using the High-Capacity cDNA Reverse Transcription Kit (Thermo Fisher Scientific; Waltham, MA, USA), and cDNA was stored at −20 °C until further analysis. Quantitative real-time PCR (RT-qPCR) was performed using an ABI 7500 FAST system (Applied Biosystems; Carlsbad, CA, USA). STAT3 expression was analyzed using SYBR™ Green PCR Master Mix (Thermo Fisher Scientific; Waltham, MA, USA), with the following primers: STAT3 forward (5′-ACCAGCAGTATAGCCGCTTC-3′) and reverse (5′-GCCACAATCCGGGCAATCT-3′). GAPDH was used as the endogenous control, with forward (5′-GCCAGCATCGCCCCACTTG-3′) and reverse (5′-GAAGGTGAAGGTCGGAGT-3′) primers. Gene expression was also evaluated using TaqMan™ Gene Expression Assays (Thermo Fisher Scientific; Waltham, MA, USA), including IL-6 (Hs00174131_m1), IDO1 (Hs00984148_m1), and NOS2 (Hs01075529_m1), with GAPDH (Hs99999905_m1), ACTB (Hs01060665_g1), or HPRT1 (Hs02800695_m1) used as endogenous controls. Relative gene expression levels were calculated using the 2^−ΔΔCt^ method [30]. Statistical analyses were performed using GraphPad Prism v10.1.2 (GraphPad Software, San Diego, CA, USA).

### 2.13. Protein Expression Analysis by Western Blotting

To investigate the modulation of signaling pathways associated with cell survival, chemoresistance, and migration, protein expression was analyzed by Western blotting in stable hGBM spheroids. After treatment, spheroids were carefully collected, washed with sterile PBS and centrifuged twice at 2000 rpm for 5 min at 4 °C to obtain a pellet free of culture medium. Total proteins were extracted on ice using lysis buffer containing 4 M urea, 2% SDS, 2 mM EGTA, 62.5 mM Tris-HCl (pH 6.8), 2 mM EDTA and 0.5% Triton X-100, supplemented with 1 µL/mL of protease inhibitor cocktail (Sigma-Aldrich; St. Louis, MO, USA). Protein concentration was determined by the Lowry method, and all experiments were performed in biological triplicates using a commercial kit (Bio-Rad; Hercules, CA, USA). For Western blot analysis, 30 µg of total protein were separated by SDS-PAGE and electrotransferred onto PVDF membranes (Bio-Rad; Hercules, CA, USA). Membranes were blocked for 1 h at room temperature with 5% non-fat milk (Molico; Araras, SP, Brazil) in TBS-T (50 mM Tris-HCl, 150 mM NaCl, 0.05% Tween-20, pH 7.4). Membranes were then incubated overnight at 4 °C with the following primary antibodies: anti-α-tubulin (mouse monoclonal; 1:1000; sc-23948; Santa Cruz Biotechnology; Dallas, TX, USA; RRID: AB_628410) as loading control; anti-STAT3 (mouse monoclonal; 1:500; sc-8019; Santa Cruz Biotechnology; Dallas, TX, USA; RRID: AB_628293); anti-PI3K C2β (rabbit polyclonal; 1:500; sc-134766; Santa Cruz Biotechnology; Dallas, TX, USA; RRID: AB_10991529); anti-Akt1/2/3 (rabbit polyclonal; 1:500; sc-8312; Santa Cruz Biotechnology; Dallas, TX, USA; RRID: AB_671714); anti-fibronectin (rabbit polyclonal, 1:1000; F3648; Sigma-Aldrich; St. Louis, MO, USA; RRID: AB_476976); and anti-MMP-2 (rabbit polyclonal; 1:500; sc-10736; Santa Cruz Biotechnology; Dallas, TX, USA; RRID: AB_2250826). Following three washes with TBS-T, membranes were incubated for 1 h at room temperature with HRP-conjugated anti-mouse or anti-rabbit secondary antibodies (1:5000; Molecular Probes; Eugene, OR, USA). Protein bands were detected using enhanced chemiluminescence substrate (ECL Plus Bio-Rad substrate kit; Hercules, CA, USA) and captured using an ImageQuant LAS 500 imaging system (GE Healthcare Life Sciences; Marlborough, MA, USA). Band intensities were quantified by densitometry using FIJI/ImageJ 1.54p software (National Institutes of Health; Bethesda, MD, USA), and protein expression levels were normalized to α-tubulin and expressed as fold change relative to control. Data represent the mean of three independent experiments.

### 2.14. Quantification of Reactive Oxygen Species (ROS)

Human GBM cell lines GL15 and U343 were cultured to generate spheroids containing approximately 3375 cells per spheroid. Spheroids were exposed to rutin 15 μM, TMZ 500 μM, rutin combined with TMZ, vehicle control DMSO 0.5%, or fresh medium. After 72 h, spheroids maintained in fresh medium were exposed to 100 μM H_2_O_2_ for 1 h and used as a positive control for ROS production. Following exposure, spheroids were collected and resuspended in PBS. The suspension contained approximately 81 spheroids in 2 mL. For the ROS assay, the suspension was distributed into black 96-well plates. Each well received 195 μL of spheroid suspension and 5 μL of the fluorescent probe DCFH-DA (D6883; Sigma-Aldrich; St. Louis, MO, USA), resulting in a final volume of 200 μL and a final concentration of 10 μM. Plates were incubated for 30 min at 37 °C protected from light. Fluorescence was measured using a microplate reader (Varioskan™ LUX multimode microplate reader; Thermo Fisher Scientific; Waltham, MA, USA) with an excitation wavelength of 485 nm and an emission wavelength of 520 nm. All experiments were performed in biological triplicates.

### 2.15. Statistical Analysis

Experimental data are presented as mean ± standard error of the mean (SEM). Statistical analyses were conducted using GraphPad Prism v10.1.2 (GraphPad Software; San Diego, CA, USA). Data distribution was assessed using the Shapiro–Wilk test. Differences among groups were evaluated using one-way analysis of variance (ANOVA) followed by Dunnett’s multiple comparisons test. Comparisons were performed relative to the vehicle control (DMSO), indicated by asterisks (*), and to the TMZ-treated group, indicated by hashtags (#). A *p* value < 0.05 was considered statistically significant. All experiments were performed in at least three independent biological replicates. For spheroid-based analyses, at least eight individual spheroids per condition were evaluated in each experiment and averaged to generate a single value per biological replicate (*n* = 3).

## 3. Results

### 3.1. Rutin Enhances TMZ Cytotoxicity

**Figure 1:**
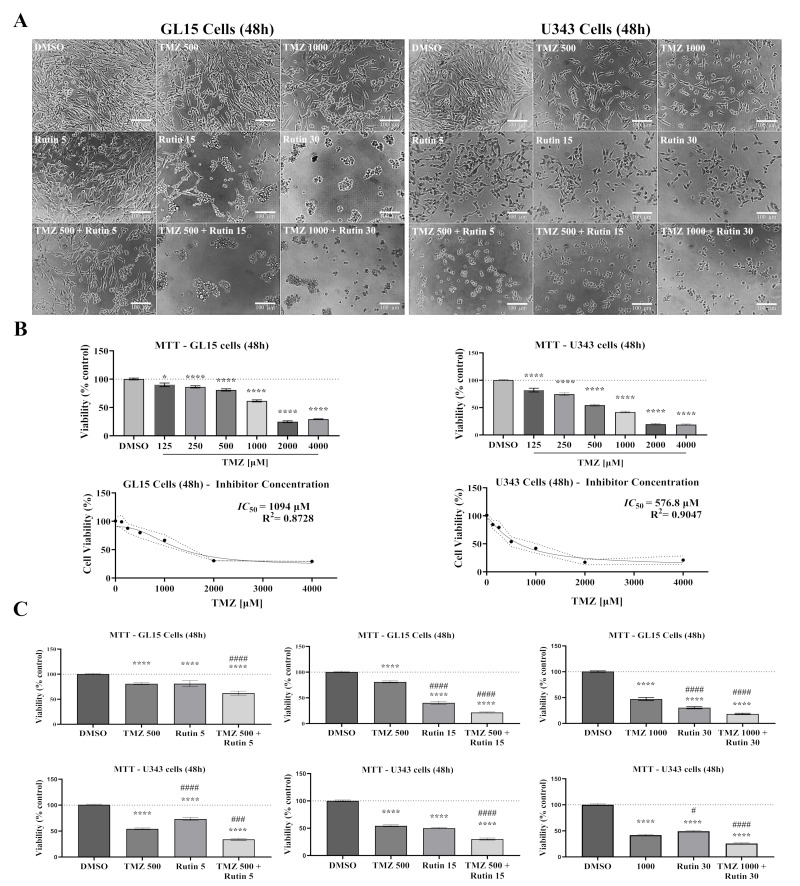
Effect of the flavonoid rutin and/or the chemotherapy drug TMZ on the viability of hGBM cells cultured in monolayer (2D). The GL15 and U343 cell lines were exposed to different concentrations of TMZ (125–4000 μM), rutin (5–30 μM), or combinations (TMZ + rutin) for 48 h, compared to the control cultures (0.5–1% DMSO). (**A**) Representative phase-contrast photomicrographs showing the density and morphological changes induced by the treatments in GL15 and U343 cultures; scale bar = 100 μm. (**B**) Cell viability analysis by the MTT assay demonstrating the dose-dependent cytotoxic effect of TMZ and the IC_50_ values for each cell line. (**C**) Bar graphs showing cell viability following combined treatments with TMZ and rutin (TMZ 500 µM + rutin 5 µM, TMZ 500 µM + rutin 15 µM, or TMZ 1000 µM + rutin 30 µM) in both cell lines. Relative cell viability (% of control) are expressed as mean ± SEM (*n* = 3). Statistical analysis: One-way ANOVA followed by Dunnett’s multiple comparison test. Asterisks (*) indicate significance relative to the control group (DMSO), whereas hashtags (#) indicate significance relative to the TMZ-treated group. Significant differences are represented as * *p* < 0.05 and **** *p* < 0.0001 vs. DMSO or # *p* < 0.05 vs. TMZ, ### *p* < 0.001 vs. TMZ and #### *p* < 0.0001 vs. TMZ.

First, to investigate whether rutin modulates GBM cell viability and enhances TMZ-induced cytotoxicity, GL15 and U343 cells in monolayer cultures were exposed for 48 h to TMZ (125–4000 µM), rutin (5–30 µM), or their combination, or maintained under control conditions (DMSO), and cell viability was assessed in 2D cultures using the MTT assay, which monitors metabolic activity, and morphological changes were monitored by phase-contrast microscopy (Figure 1). In GL15 cell cultures, low concentrations of TMZ (125–250 µM) did not induce noticeable morphological alterations compared to control cultures, with cells maintaining fusiform morphology and adherence, although a modest reduction in viability was observed. At 500 µM TMZ, viability decreased to 80.98%, followed by a concentration-dependent reduction in cell density. From 1000 µM onward, a more pronounced decrease in viability (47.21%) was accompanied by an increased prevalence of shrunken and rounded cells. The IC_50_ value for TMZ was 1094 µM, indicating relative resistance of this cell line to chemotherapy. In contrast, the U343 cell line exhibited greater sensitivity to TMZ exposure. At 500 µM TMZ, viability decreased to 54.42%, accompanied by reduced cell density and the presence of shrunken cells (*p* < 0.0001 vs. DMSO). This effect intensified with increasing concentrations, yielding an IC_50_ of 576.8 µM (Figure 1B). Rutin (5–30 µM) exerted a dose-dependent cytotoxic effect in both GBM cell lines. At 5 µM, viability was 81.01% in GL15 and 73.29% in U343 (*p* < 0.0001 vs. DMSO). At higher concentrations (15–30 µM), viability was progressively reduced, reaching 40.29% and 30.40% in GL15, and 50.14% and 49.26% in U343. These effects were accompanied by morphological changes, including cell shrinkage and rounding. Notably, the combination of rutin with TMZ enhanced cytotoxicity in both cell lines. At 5 µM rutin combined with 500 µM TMZ, viability decreased to 62.19% in GL15 and 34.01% in U343 (*p* < 0.0001 vs. TMZ). Higher concentrations further enhanced this effect, reducing viability to 21.45% and 30.19% at 15 µM rutin + 500 µM TMZ, and to 18.39% and 25.79% at 30 µM rutin combined with 1000 µM TMZ. These conditions were associated with marked loss of confluence, increased cell detachment, and near-complete loss of adhesion at the highest concentrations (*p* < 0.0001 vs. DMSO; Figure 1A,C).

### 3.2. Rutin Is Not Toxic to Astrocytes, Presenting Selectivity to Glioblastoma Cells

**Figure 2:**
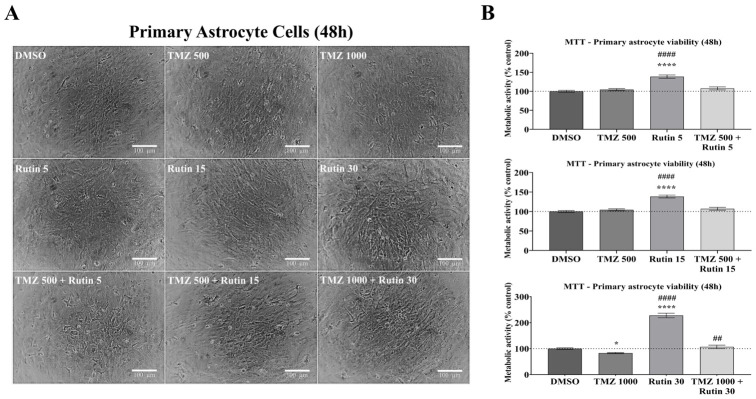
Effect of rutin and/or temozolomide (TMZ) on the viability of primary astrocytes derived from mouse (P0–P2) cultured in monolayer (2D). Primary astrocytes were exposed to different concentrations of TMZ (500–1000 μM), rutin (5–30 μM), or their combinations (TMZ + rutin) for 48 h, compared to control cultures (1% DMSO). (**A**) Representative phase-contrast photomicrographs showing cell density and morphological features under the different treatment conditions; scale bar = 100 μm. (**B**) Cellular metabolic activity assessed by the MTT assay showing the effects of rutin, TMZ, and their combinations (TMZ 500 μM or 1000 μM combined with rutin 5–30 μM). Relative metabolic activity (% of control) is expressed as mean ± SEM (*n* = 3). Statistical analysis was performed using one-way ANOVA followed by Dunnett’s multiple comparison test. Asterisks (*) indicate significance relative to the control group (DMSO), whereas hashtags (#) indicate significance relative to the TMZ-treated group. Significant differences are represented as * *p* < 0.05, **** *p* < 0.0001 vs. DMSO, or ## *p* < 0.01 and #### *p* < 0.0001 vs. TMZ 1000.

Given that rutin enhanced TMZ-induced cytotoxicity in hGBM cell lines (GL15 and U343), derived from grade IV astrocytomas [1], we investigated whether this effect is selective by evaluating its impact on non-tumoral astrocytes. Primary astrocytes derived from the cortex of postnatal mice (P0–P2) were cultured under 2D conditions until stabilization and then exposed for 48 h to rutin (5–30 µM), TMZ (500–1000 µM), or their combination (rutin + TMZ), with control cells maintained in DMSO. Cellular metabolic activity was assessed using the MTT assay, and morphological changes were analyzed by phase-contrast microscopy (Figure 2). Phase-contrast analysis revealed no evident alterations in cell density or morphology under any experimental condition, with astrocytes maintaining their adherent morphology and confluence throughout treatments (Figure 2A). Treatment with TMZ at 500 µM did not significantly affect cellular metabolic activity compared to control, whereas exposure to TMZ at 1000 µM resulted in an approximately 17% reduction in metabolic activity (*p* < 0.05 vs. DMSO; Figure 2B). Treatment with rutin alone increased cellular metabolic activity after 48 h, with concentrations of 5 µM and 15 µM promoting an approximately 38% increase compared to control (*p* < 0.0001 vs. DMSO), while 30 µM induced an approximately 128% increase (*p* < 0.0001 vs. DMSO). In the combined treatment of rutin (30 µM) and TMZ (1000 µM), the reduction in metabolic activity of astrocytes was no longer evident, with values significantly higher than those observed with TMZ alone (*p* < 0.01 vs. TMZ 1000 µM).

### 3.3. Rutin Modulates hGBM 3D Spheroid Tumor Formation and Enhances TMZ Toxicity

**Figure 3:**
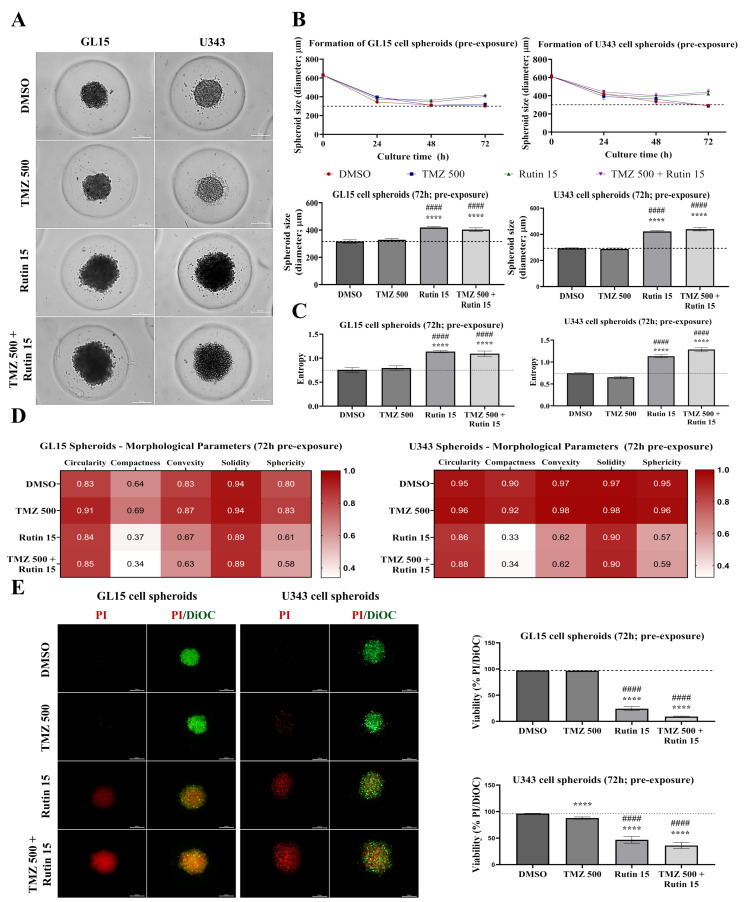
Effect of the flavonoid rutin and/or the chemotherapy drug TMZ on the formation, morphology, and viability of hGBM spheroids derived from GL15 and U343 cells. Spheroids were cultured for 72 h in the presence of rutin (15 μM), TMZ (500 μM), a combination of both, or maintained under control conditions (0.5% DMSO). (**A**) Representative phase-contrast microscopy images showing spheroid formation and compaction. (**B**) Temporal evolution of the mean diameter over 3 days of culture, with comparative quantification on day 3. (**C**) Entropy analysis, evidencing changes in peripheral organization and spheroidal compaction pattern. (**D**) Heat map of spheroid morphometric parameters. Circularity, compactness, convexity, solidity, and sphericity were extracted using AnaSP software, with values ranging from 0 to 1. Higher values in red indicate increased spheroid organization and compaction, whereas lower values in white reflect reduced structural integrity. (**E**) Representative fluorescence images showing total cells (DiOC_18_(3), green) and nonviable/necrotic cells (PI, red) in the different treatments. Relative cell viability was calculated from the PI/DiOC_18_(3) fluorescence ratio and normalized to control conditions. Intensity analysis was conducted using Fiji/ImageJ software (v1.54p). Results are presented as mean ± standard error of the mean (SEM) of three independent biological experiments (*n* = 3). Statistical significance was determined by one-way ANOVA followed by Dunnett’s multiple comparison test. Asterisks (*) indicate significance relative to the control group (DMSO), whereas hashtags (#) indicate significance relative to the TMZ-treated group. Significant differences are represented as **** *p* < 0.0001 vs. DMSO or #### *p* < 0.0001 vs. TMZ. Scale bar = 200 μm.

Following the observed effects of rutin on hGBM cell viability and TMZ sensitivity in 2D cultures, we next evaluated its impact in a 3D spheroid model, which better recapitulates tumor architecture, cell–cell interactions, and treatment resistance [31]. To this end, morphology and viability were assessed during spheroid formation using sub-IC_50_ concentrations. GL15 and U343 cells were cultured in the presence of TMZ (500 μM), rutin (15 μM), or their combination for 72 h. Cells were exposed to the compounds from the onset of culture, allowing dynamic assessment of aggregation and spheroid architecture throughout the experimental period. Under control conditions (0.5% DMSO) or TMZ treatment, spheroids displayed similar diameters, as well as comparable peripheral structural features and entropy (Figure 3). In these conditions, cells from both GBM cell lines retained the ability to form compact spheroids, with similar circularity, compactness, convexity, solidity, and sphericity. In contrast, rutin-treated groups exhibited a significant increase in spheroid diameter from 48 h onward compared with control, accompanied by increased entropy (*p* < 0.0001 vs. control; *p* < 0.0001 vs. TMZ; Figure 3A–C). Rutin exposure progressively altered spheroid organization over 72 h. Quantitative analysis revealed increased entropy and edge irregularity, associated with reduced circularity and compaction (Figure 3A,D). Fluorescence-based viability analysis (PI/DiOC_18_(3)) demonstrated that GL15 spheroids maintained viability comparable to control following TMZ exposure. In contrast, U343 spheroids showed a reduction in viability to 87.85% upon TMZ exposure (*p* < 0.0001 vs. control). In spheroids from both cell lines, rutin alone reduced viability to 24.16% in GL15 and 46.79% in U343 (*p* < 0.0001 vs. control), and, when combined with TMZ, reduced viability to 9.09% in GL15 and 36.11% in U343 (*p* < 0.0001 vs. TMZ; Figure 3E).

### 3.4. Rutin Enhances TMZ and Modulates Survival and Chemoresistance Regulators in Established hGBM Spheroids

**Figure 4:**
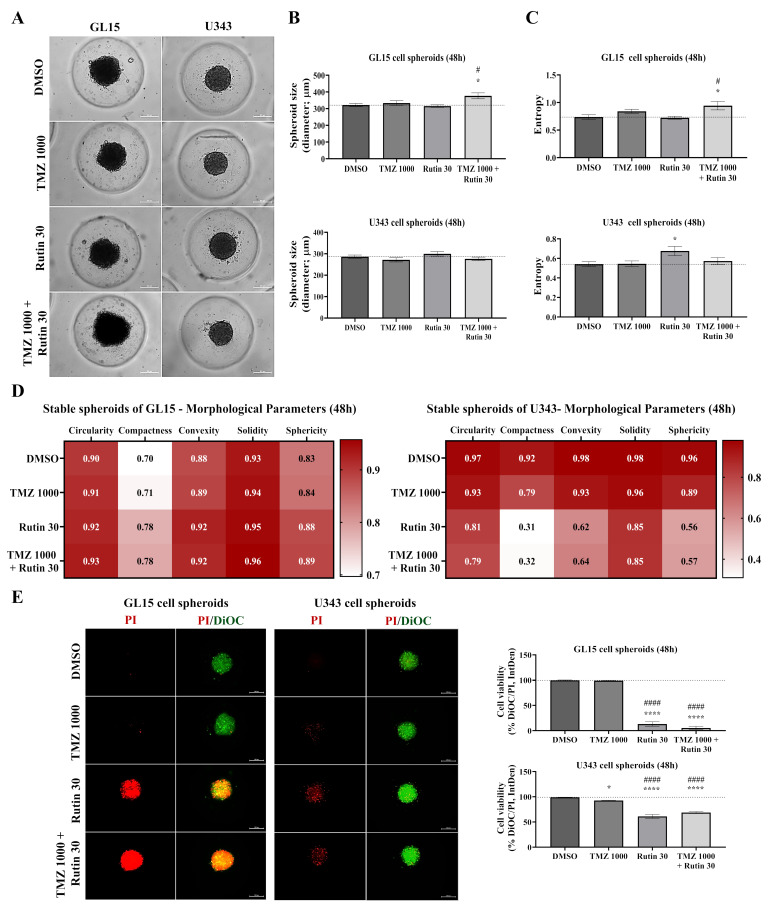
Effect of the flavonoid rutin and/or the chemotherapy agent TMZ on the morphology, viability, and protein expression of stable hGBM spheroids derived from the GL15 and U343 cell lines. Cells were cultured in a 3D model for 7 days to allow the formation of stable spheroids and then exposed for 48 h to rutin (30 μM), TMZ (1000 μM), their combination, or control conditions (1% DMSO). (**A**) Representative phase-contrast images showing spheroid morphology after treatments. (**B**) Mean spheroid diameter after 48 h of treatment. (**C**) Entropy analysis indicating changes in spheroid organization and compaction. (**D**) Heat map of spheroid morphometric parameters. Circularity, compactness, convexity, solidity, and sphericity were extracted using AnaSP software, with values ranging from 0 to 1. Higher values in red indicate increased spheroid organization and compaction, whereas lower values in white reflect reduced structural integrity. (**E**) Representative fluorescence images showing viable cells stained with DiOC_18_(3) (green) and nonviable/necrotic cells labeled with propidium iodide (PI, red). Relative cell viability was calculated from the PI/DiOC_18_(3) fluorescence ratio and normalized to control conditions. Data are presented as mean ± SEM from three independent biological experiments (*n* = 3). Statistical analysis was performed using one-way ANOVA followed by Dunnett’s multiple comparison test. Asterisks (*) indicate significance relative to the control group (DMSO), whereas hashtags (#) indicate significance relative to the TMZ-treated group. Significant differences are indicated as * *p* < 0.05 and **** *p* < 0.0001 vs. DMSO or # *p* < 0.05 and #### *p* < 0.0001 vs. TMZ. Scale bar = 200 μm.

After evaluating the effects of rutin during spheroid formation, we examined its impact in established hGBM spheroids, which represent a more structurally stable and treatment-resistant spheroidal model. GL15 and U343 cells were cultured for 7 days to allow the formation of compact spheroids, which were then exposed for 48 h to TMZ, rutin, their combination, or control conditions (DMSO). Morphological features were assessed by phase-contrast microscopy and computational morphometric analysis (AnaSP), while cell viability was evaluated by fluorescence staining using PI (non-viable cells) and DiOC_18_(3) (total cells), allowing integrated analysis of structural and viability-related parameters (Figure 4). Under control conditions, GL15 spheroids exhibited a less regular architecture compared with U343 spheroids. Phase-contrast microscopy showed that TMZ alone preserved spheroid morphology in both cell lines. In contrast, rutin increased spheroid diameter in GL15 under combination treatment (*p* < 0.05 vs. DMSO), whereas no significant changes were observed in U343 (Figure 4A,B). Analysis of peripheral integrity revealed increased entropy in GL15 spheroids only under combined treatment (*p* < 0.05 vs. DMSO; Figure 4A,C), whereas U343 spheroids showed reduced entropy following rutin alone (*p* < 0.05 vs. DMSO; Figure 4A,C). Morphometric analysis further indicated that rutin, alone or combined with TMZ, increased circularity, compactness, convexity, solidity, and sphericity in GL15-derived spheroids, suggesting enhanced structural compaction, while the opposite trend was observed in U343 (Figure 4D). Fluorescence analysis revealed distinct viability patterns. Control spheroids showed a predominance of viable cells. TMZ resulted in a modest increase in PI labeling in U343 spheroids, corresponding to 92.63% viability. Rutin, alone or combined with TMZ, further increased PI labeling in both models, with a more pronounced effect in GL15 cells, reducing viability to 13.12% in GL15 and 61.09% in U343 under rutin alone, and to 5.20% in GL15 and 68.65% in U343 under combined treatment (Figure 4E). Quantification of fluorescence signals confirmed that, in GL15 spheroids, rutin significantly reduced cell viability compared to control (*p* < 0.0001 vs. DMSO), with a greater reduction observed under combined treatment compared to both control and TMZ alone (*p* < 0.0001 vs. TMZ).

### 3.5. Rutin Suppresses Migration of hGBM Spheroids Independently of ECM Upregulation

**Figure 5:**
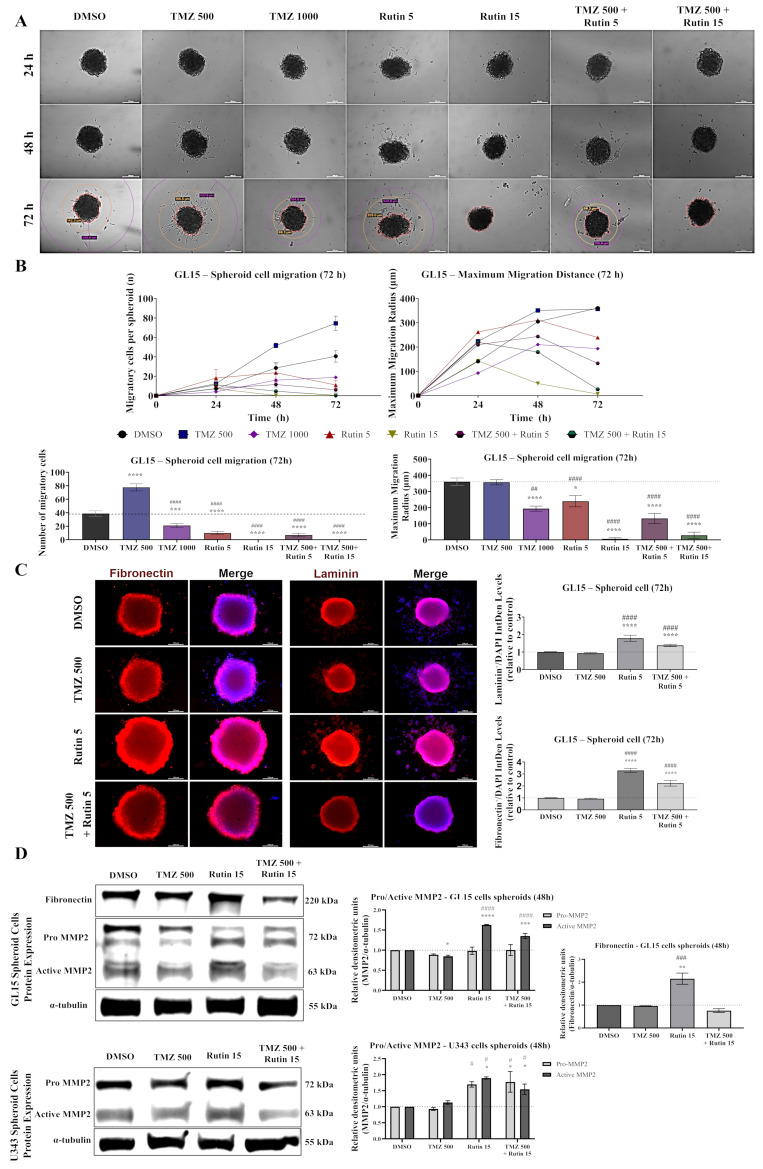
Effect of the flavonoid rutin and/or the chemotherapy agent TMZ on the migration and adhesion-related proteins of cells derived from hGBM spheroids. GL15 and U343 spheroids were generated in agarose-based 3D molds and exposed to rutin (5–15 μM), TMZ (500 or 1000 μM), their combination, or control conditions (0.5% DMSO) for 72 h. Individual spheroids were then transferred to adherent culture plates to evaluate the migratory behavior of spheroid-derived cells. (**A**) Representative phase-contrast images showing cell migration from GL15 spheroids over time following treatments; scale bar = 200 μm. (**B**) The number of migrating cells and the migratory radius from the spheroid edge were quantified. (**C**) Representative immunofluorescence images of fibronectin and laminin expression in GL15 spheroids after treatments. Fibronectin or laminin are shown in red, and nuclei are stained in blue. Merged images illustrate the distribution of extracellular matrix proteins in treated spheroids, along with the corresponding quantification of fluorescence intensity; scale bar = 100 μm. (**D**) Fibronectin and MMP2 (pro and active forms) protein expression was analyzed by Western blotting in hGBM spheroids 48 h after treatment. Representative bands are shown alongside densitometric quantification. Band intensity was quantified using FIJI/ImageJ v1.54p, normalized to α-tubulin, and expressed as fold change relative to control. Data are presented as mean ± SEM from three independent biological experiments (*n* = 3). Statistical analysis was performed using one-way ANOVA followed by Dunnett’s multiple comparison test. Asterisks (*) indicate significance relative to the control group (DMSO), whereas hashtags (#) indicate significance relative to the TMZ-treated group. Significant differences are indicated as * *p* < 0.05, ** *p* < 0.01, *** *p* < 0.001 and **** *p* < 0.0001 vs. DMSO or # *p* < 0.05, ## *p* < 0.01, ### *p* < 0.001 and #### *p* < 0.0001 vs. TMZ.

Considering the effects of rutin on established hGBM spheroids, we evaluated cell migration as an indicator of invasive potential and ECM remodeling by analyzing MMP2, fibronectin, and laminin, key regulators of matrix organization and tumor–microenvironment interactions associated with GBM progression and chemoresistance. Stable GL15 spheroids were transferred to adherent culture plates and subsequently treated with TMZ (500 or 1000 µM), rutin (5–15 µM), or their combination, or maintained under control conditions (DMSO) for up to 72 h. Spheroid-derived cells were monitored over time to assess migratory behavior, including the number of migrating cells and migration distance, while ECM-related proteins were evaluated by immunofluorescence and Western blot analysis (Figure 5). TMZ treatment exerted dose-dependent effects on cell migration. After 48 h, an increased number of migratory cells was observed in spheroids exposed to 500 µM TMZ (*p* < 0.0001 vs. DMSO), whereas a reduction in migrating cells was noted at 1000 µM (Figure 5A,B). Consistently, exposure to 500 µM TMZ did not significantly alter the migration radius compared with control conditions, while treatment with 1000 µM TMZ significantly reduced migration (*p* < 0.01 vs. DMSO; Figure 5A,B). In contrast, rutin exerted a pronounced antimigratory effect. All conditions containing rutin significantly reduced the number of migrating cells compared with TMZ-treated groups (*p* < 0.0001 vs. TMZ). Notably, at 15 µM, rutin completely abolished migratory activity after 72 h. Consistently, combined treatment with TMZ (500 µM) and rutin further suppressed cell migration (Figure 5A,B). Immunofluorescence analysis did not reveal significant changes in fibronectin or laminin expression compared to TMZ-treated conditions; however, exposure to rutin at 5 µM resulted in a significant increase in both ECM proteins (*p* < 0.0001 vs. DMSO; *p* < 0.0001 vs. TMZ; Figure 5C). Finally, we evaluated whether treatments alone or combined modulated the expression of ECM-related proteins. Fibronectin expression was increased in GL15 hGBM cells treated with rutin alone compared to control and TMZ-treated conditions (*p* < 0.01 vs. DMSO; *p* < 0.001 vs. TMZ; Figure 5D). Additionally, TMZ exposure led to a significant reduction in active MMP2 levels compared to control in GL15 spheroids (*p* < 0.05 vs. DMSO), without affecting U343 cells. In contrast, rutin exposure increased active MMP2 levels in GL15 (*p* < 0.0001 vs. DMSO; *p* < 0.0001 vs. TMZ) and increased both pro-MMP2 and active MMP2 in U343 spheroids (*p* < 0.05 vs. DMSO; *p* < 0.05 vs. TMZ; Figure 5C). Similarly, in U343 spheroids, rutin exposure increased the expression of both pro-MMP2 and active MMP2. However, unlike GL15 spheroids, U343 spheroids did not exhibit detectable migratory activity or fibronectin expression.

### 3.6. Rutin Modulates Inflammation, Chemoresistance-Associated Pathways, Kynurenine Metabolism and Redox Balance in hGBM Spheroids

**Figure 6:**
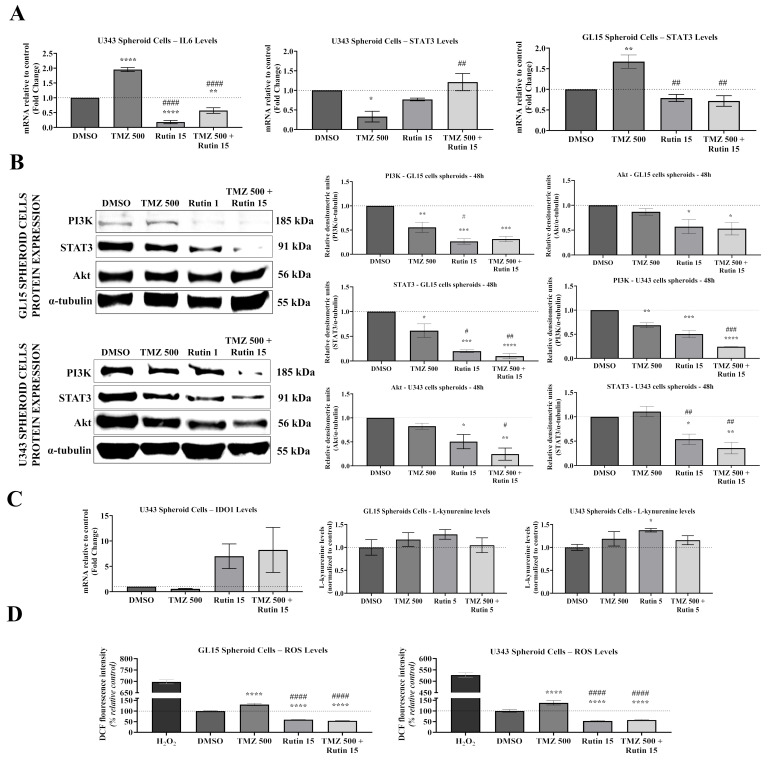
Effect of the flavonoid rutin and/or TMZ on molecular, metabolic and redox modulation in hGBM spheroids. 3D spheroids derived from hGBM cell lines GL15 and U343 were generated using micromold culture systems and exposed to rutin (15 μM), TMZ (500 μM), the combination (TMZ + rutin), or vehicle control (0.5% DMSO). For molecular analyses, stable spheroids were exposed to the treatments for 48 h, whereas metabolic and redox parameters were evaluated during spheroid formation after 72 h of exposure. (**A**) Relative mRNA expression of IL-6 and STAT3 was determined by RT-qPCR and normalized to lineage-specific endogenous controls (β-tubulin for GL15 and GAPDH for U343). (**B**) Protein expression of STAT3, PI3K, and Akt was analyzed by Western blotting in stable spheroids after 48 h of treatment. Representative bands with corresponding molecular weights are shown alongside densitometric quantification. Band intensity was quantified using FIJI/ImageJ (v1.54p), normalized to α-tubulin, and expressed as fold change relative to control. (**C**) Evaluation of the kynurenine pathway through IDO1 mRNA expression and quantification of its metabolic product L-kynurenine, determined by colorimetric assay. (**D**) Intracellular reactive oxygen species (ROS) production during spheroid formation after 72 h of exposure was determined using the DCFH-DA (2′,7′-dichlorodihydrofluorescein diacetate) assay, in which intracellular oxidation of the probe generates the fluorescent compound DCF, proportional to ROS levels. Data are presented as the mean ± standard error of the mean (SEM) of three biological replicates (*n* = 3). Statistical significance was determined by one-way ANOVA followed by Dunnett’s multiple comparison test. Asterisks (*) indicate significance relative to the control group (DMSO), whereas hashtags (#) indicate significance relative to the TMZ-treated group. Significant differences are represented as * *p* < 0.05, ** *p* < 0.01, *** *p* < 0.001 and **** *p* < 0.0001 vs. DMSO or # *p* < 0.05, ## *p* < 0.01, ### *p* < 0.001 and #### *p* < 0.0001 vs. TMZ.

Based on the effects of rutin alone and in association with TMZ on cell migration and ECM remodeling in hGBM spheroids, we next investigated whether these phenotypic changes are associated with modulation of tumor-related signaling immunometabolic pathways, and key regulators of adaptive responses involved in GBM progression and chemoresistance, as the redox balance, defined by intracellular ROS levels, and kynurenine production, as a readout of IDO activity. For this, 3D spheroids were generated from the hGBM cell lines GL15 and U343. Stable spheroids were exposed to rutin (15 μM), TMZ (500 μM), their combination, or vehicle control (0.5% DMSO). Gene expression analyses were performed by RT-qPCR, while protein expression was assessed by Western blot after 48 h of treatment. Intracellular ROS levels were quantified using the DCFH-DA fluorescence assay, whereas L-kynurenine levels were determined by a colorimetric assay in culture supernatants, with metabolic and redox parameters evaluated during spheroid formation after 72 h of treatment (Figure 6).

To determine whether rutin modulates inflammatory signaling associated with chemoresistance, we evaluated the IL-6/STAT3 axis in GBM spheroids, a signaling pathway associated with tumor progression and inflammatory regulation [32]. In U343 spheroids, TMZ exposure significantly increased IL-6 mRNA levels compared with the control group (*p* < 0.0001 vs. DMSO). However, rutin alone and in combination with TMZ, significantly reduced IL-6 expression compared to DMSO (*p* < 0.0001 vs. DMSO) and compared to TMZ (*p* < 0.0001 vs. TMZ; Figure 6A). STAT3 transcription was also modulated by the treatments. In U343 spheroids, TMZ exposure led to a significant reduction in STAT3 mRNA expression compared with control (*p* < 0.05 vs. DMSO); however, this effect was reversed in the presence of rutin, with increased STAT3 expression in the combined treatment (*p* < 0.01 vs. TMZ; Figure 6A). In GL15 spheroids, TMZ treatment significantly increased STAT3 expression compared to control (*p* < 0.01 vs. DMSO). In contrast, rutin exposure significantly reduced STAT3 expression compared to TMZ treatment (*p* < 0.01 vs. TMZ; Figure 6A). Notably, IL-6 mRNA expression was not detected in GL15 spheroids under any of the experimental conditions.

Given that IL-6/STAT3 signaling functionally interacts with the PI3K/Akt pathway to promote tumor survival and chemoresistance [33], we investigated whether these transcriptional alterations were reflected at the protein level by assessing STAT3, PI3K, and Akt expression in GBM spheroids. In GL15 spheroids, STAT3 protein levels were significantly reduced following TMZ exposure compared to DMSO (*p* < 0.05). This reduction was further enhanced by rutin treatment, both alone and in combination with TMZ. Rutin reduced STAT3 levels compared to TMZ (*p* < 0.05 vs. TMZ). The combination further reduced STAT3 compared to TMZ (*p* < 0.01 vs. TMZ) and compared to DMSO (*p* < 0.0001 vs. DMSO; Figure 6B). In U343 spheroids, TMZ did not markedly alter STAT3 protein levels compared to DMSO, although a tendency toward increased expression was observed. However, rutin treatment significantly reduced STAT3 expression when administered alone (*p* < 0.05 vs. DMSO; *p* < 0.01 vs. TMZ) and in combination with TMZ (*p* < 0.01 vs. DMSO; *p* < 0.01 vs. TMZ). In both GL15 and U343 spheroids, PI3K protein levels were reduced following TMZ exposure compared to DMSO (*p* < 0.01 vs. DMSO). Rutin treatment reduced PI3K expression in both cell lines compared to DMSO (*p* < 0.001 vs. DMSO). In GL15 spheroids, rutin decreased PI3K levels relative to TMZ (*p* < 0.05 vs. TMZ). In U343 spheroids, both rutin and the combined treatment reduced PI3K expression compared to DMSO (*p* < 0.001 vs. DMSO; *p* < 0.0001 vs. DMSO), while the combination further decreased PI3K levels relative to TMZ (*p* < 0.0001 vs. TMZ). No significant changes in Akt protein levels were observed following TMZ treatment alone in either cell line. In contrast, rutin reduced Akt expression compared to DMSO (*p* < 0.05 vs. DMSO). The combined treatment further decreased Akt levels relative to TMZ in both GL15 and U343 spheroids (*p* < 0.05 vs. TMZ), with an additional reduction observed in U343 compared to DMSO (*p* < 0.01 vs. DMSO).

IDO1–KP plays an important role in tumor immunometabolism and immune evasion in GBM [34]. Hence, we evaluated whether rutin modulates this axis by assessing IDO1 expression and L-kynurenine production in GBM spheroids (Figure 6C). In U343 spheroids, IDO1 mRNA levels did not show statistically significant differences across treatments, although rutin exposure showed a tendency toward increased expression. Consistently, L-kynurenine levels remained similar across treatments in GL15 spheroids. In U343 spheroids, rutin increased L-kynurenine levels compared with control (*p* < 0.05 vs. DMSO), whereas TMZ or the combination did not show statistically significant differences (Figure 6C).

In line with the role of TMZ-induced ROS in disrupting redox homeostasis and promoting adaptive responses in GBM [4], intracellular ROS levels were evaluated in GL15 and U343 spheroids during formation using the DCFH-DA assay (Figure 6D). In both cell lines, TMZ exposure increased DCF fluorescence intensity compared to control, indicating enhanced ROS production (*p* < 0.0001 vs. DMSO). In contrast, rutin exposure reduced ROS levels when administered alone (*p* < 0.0001 vs. DMSO). Moreover, rutin significantly decreased ROS levels in spheroids subjected to combined treatment, demonstrating that rutin attenuates ROS production even in the presence of TMZ, compared with both control and TMZ-treated groups (*p* < 0.0001 vs. TMZ; Figure 6D).

### 3.7. Rutin Pretreatment Enhances TMZ Sensitivity and Modulates NO/IDO1 Signaling During hGBM Spheroid Formation

**Figure 7:**
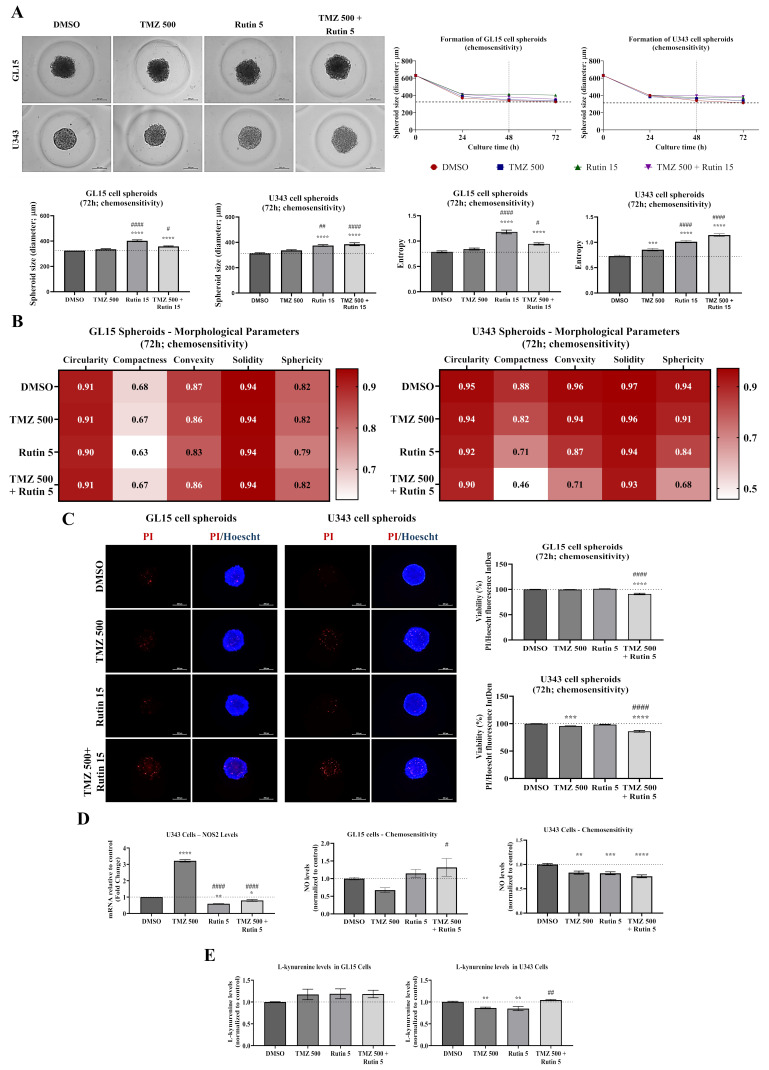
Effect of rutin pretreatment on TMZ sensitivity, nitric oxide (NO) expression and indoleamine 2,3-dioxygenase (IDO) enzymatic activity during hGBM spheroid formation. GL15 and U343 cells were seeded in agarose-based 3D molds to allow spheroid formation under experimental conditions. Cells were exposed to rutin (5 μM) for 24 h and subsequently treated with TMZ (500 μM) for an additional 48 h, totaling 72 h of treatment. Control groups received drug vehicle only (0.5% DMSO). (**A**) Representative phase-contrast images showing spheroid morphology after treatments and changes in structural organization. (**B**) Heat maps of morphometric parameters (circularity, compactness, convexity, solidity, and sphericity) obtained using AnaSP software. (**C**) Representative fluorescence images showing nonviable cells labeled with propidium iodide (PI, red) and nuclei stained with Hoechst (blue), with corresponding quantification of PI fluorescence as an indicator of cell viability. (**D**) Relative NOS2 mRNA expression, determined by RT-qPCR, and NO levels in the supernatant of GL15 and U343 cells following treatment. (**E**) IDO enzymatic activity inferred from L-kynurenine levels measured in the culture medium. Data are presented as mean ± SEM from three independent biological experiments (*n* = 3). Statistical analysis was performed using one-way ANOVA followed by Dunnett’s multiple comparison test. Significant differences are indicated as * *p* < 0.05, ** *p* < 0.01, *** *p* < 0.001 and **** *p* < 0.0001 vs. DMSO or # *p* < 0.05, ## *p* < 0.01 and #### *p* < 0.0001 vs. TMZ. Scale bar = 200 μm.

Finally, given that rutin modulated key adaptive pathways associated with chemoresistance, including redox balance, immunometabolic activity, and tumor-related signaling, we investigated whether rutin pretreatment influences hGBM cell responsiveness to TMZ, aiming to determine whether this modulation could enhance chemosensitivity. GL15 and U343 cells were seeded in 3D micromolds and pre-exposed to rutin (5 µM) for 24 h, followed by TMZ treatment (500 µM) for an additional 48 h, totaling 72 h of experimental duration, while control groups received vehicle (0.5% DMSO). Morphological parameters and texture metrics were evaluated to assess spheroid structural organization, and cell viability was determined by fluorescence staining using PI and Hoechst (Figure 7A–C).

Rutin exposure, with or without subsequent TMZ treatment, induced marked alterations in spheroid morphology and structural organization. Phase-contrast microscopy showed that TMZ treatment did not significantly affect spheroid diameter but increased entropy compared to control conditions (*p* < 0.01 vs. DMSO). More pronounced morphological changes were observed in spheroids pre-exposed to rutin in both cell lines (*p* < 0.0001 vs. DMSO; *p* < 0.0001 vs. TMZ; Figure 7A), including increased entropy and irregular spheroid borders, indicating reduced structural homogeneity. At 72 h, rutin alone increased spheroid diameter in both GL15 (*p* < 0.0001 vs. DMSO; *p* < 0.05 vs. TMZ) and U343 (*p* < 0.0001 vs. DMSO; *p* < 0.01 vs. TMZ), while the combination with TMZ further increased diameter (*p* < 0.0001 vs. DMSO; *p* < 0.05 vs. TMZ). Additionally, pre-exposure to rutin, either alone or in combination with TMZ, increased structural entropy in both cell lines compared to control and TMZ alone (*p* < 0.0001 vs. DMSO; *p* < 0.05 vs. TMZ; *p* < 0.0001 vs. DMSO). Consistently, morphometric heatmap analysis demonstrated alterations in circularity, compactness, convexity, solidity, and sphericity across treatment groups (Figure 7B), with minor variations in GL15 and more pronounced reductions in U343 spheroids when TMZ was administered after rutin pretreatment. Fluorescence analysis using PI and Hoechst staining revealed low levels of cell death under control conditions, whereas TMZ increased the number of PI-positive cells in U343 spheroids. Rutin pretreatment increased PI-positive cells in both GL15 and U343 spheroids upon TMZ exposure (*p* < 0.0001 vs. DMSO; *p* < 0.0001 vs. TMZ; Figure 7C). In agreement, viability analysis showed that the combination treatment significantly reduced GL15 cell viability (*p* < 0.0001 vs. DMSO; *p* < 0.0001 vs. TMZ). In U343 spheroids, TMZ alone reduced viability compared to control (*p* < 0.001 vs. DMSO), and this effect was further enhanced in cells pretreated with rutin (*p* < 0.0001 vs. DMSO; *p* < 0.0001 vs. TMZ).

Moreover, NO levels and NOS2 expression were evaluated as indicators of redox and inflammatory signaling, while kynurenine levels were used to infer IDO-mediated immunometabolic activity (Figure 7D–E). In U343 spheroids, RT-qPCR analysis showed that TMZ treatment significantly increased NOS2 expression compared with control conditions (*p* < 0.0001 vs. DMSO). Rutin pre-exposure markedly reduced NOS2 expression, both alone and in combination with TMZ, compared with control and TMZ-treated groups (*p* < 0.001 vs. DMSO; *p* < 0.0001 vs. TMZ; Figure 7D). Rutin alone also reduced NOS2 expression (*p* < 0.01 vs. DMSO; *p* < 0.0001 vs. TMZ). NO levels in the supernatant showed distinct responses between cell lines. In GL15 spheroids, NO levels tended to decrease following TMZ treatment and increase after rutin pre-exposure, with a significant increase observed in the combination group (*p* < 0.05 vs. DMSO). In U343 spheroids, all treatments reduced NO levels compared with control conditions, with the most pronounced decrease observed when TMZ was administered after rutin pretreatment (*p* < 0.01 vs. DMSO; *p* < 0.001 vs. DMSO; *p* < 0.0001 vs. DMSO). L-kynurenine levels also exhibited cell line–dependent effects. In GL15 spheroids, all treatments showed a tendency toward increased kynurenine levels compared to control. In U343 spheroids, treatment with TMZ or rutin alone reduced kynurenine levels relative to control (*p* < 0.01 vs. DMSO), whereas kynurenine levels were significantly increased after TMZ treatment following rutin pretreatment compared to TMZ alone (*p* < 0.01 vs. TMZ; Figure 7E).

## 4. Discussion

Although TMZ constitutes the main chemotherapeutic agent in GBM treatment, tumor cells frequently develop chemoresistance driven by adaptive responses involving the tumor microenvironment, redox imbalance, and pro-survival signaling, promoting tumor persistence and immunosuppression [14]. In this context, although the cytotoxicity and modulation of signaling pathways by rutin in 2D GBM models have already been described, in this study we investigated whether rutin, in combination with TMZ, is capable of modulating the redox status, pathways associated with chemoresistance, and migratory potential in more complex models, such as hGBM spheroids.

Thus, we evaluated whether rutin enhances TMZ-induced cytotoxicity at sub-IC_50_ concentrations. Our results showed that rutin enhances the cytotoxic effects of TMZ even at low doses, whereas previous studies have reported cytotoxic effects of rutin only at higher concentrations (30–50 µM) [17,18]. Here, in 2D culture, the combination of rutin (5 µM) with TMZ (500 µM), even at the lowest concentration tested, reduced cell viability from 80.98% to 62.19% in GL15 and from 54.42% to 34.01% in U343, highlighting the greater resistance of GL15 cells to TMZ. These findings are consistent with previous studies showing that rutin inhibits treatment-induced cytoprotective autophagy, a key adaptive mechanism in GBM cell survival [35], and with evidence highlighting the role of multiple adaptive resistance pathways that support the use of combination therapies in GBM [13]. Although our findings indicate enhanced efficacy under combined treatment conditions, no formal drug interaction analysis was performed. Therefore, the observed effects should still be interpreted as additive or potentially cooperative, rather than definitively synergistic. In addition to its effects in GBM cells, we evaluated the impact of rutin on non-tumoral astrocytes, given that GBM is a grade IV astrocytoma of astrocytic origin [1]. Rutin increased astrocyte metabolic activity, as measured by the MTT assay, and attenuated the reduction induced by TMZ. This effect is consistent with the intrinsic metabolic role of astrocytes, which are actively involved in energy homeostasis, and with previous evidence showing that flavonoids can modulate cellular metabolism and mitochondrial function in non-tumoral cells [36]. In line with this, plant-derived compounds have been shown to directly affect mitochondrial function and cellular bioenergetics in tumor cells, highlighting metabolic vulnerabilities as potential therapeutic targets [37]. Taken together, these findings contrast with the toxicity to GBM cells and reinforce the differential responses between tumor and non-tumoral cells. Considering the limitations of 2D systems and the metabolic nature of the MTT assay, three-dimensional models and co-culture approaches may further elucidate astrocyte–tumor interactions in this context.

Given the results obtained in 2D cultures, treatment responses were further evaluated in spheroids, which better reflect spatial tumor organization. In these models, limited drug penetration can lead to heterogeneous exposure across the structure, thereby altering therapeutic efficacy compared to monolayer cultures [38]. To characterize treatment-induced alterations in spheroid architecture, radiomic descriptors, including diameter, circularity, compactness, convexity, sphericity, solidity, and entropy, were analyzed alongside cell viability. More compact and regular spheroids are associated with increased cell–cell adhesion and treatment resistance, while reductions in these metrics indicate structural disruption and increased susceptibility to therapy [39]. Entropy complements these descriptors by capturing structural heterogeneity. Increased entropy reflects spatial disorganization and reduced architectural integrity, potentially promoting the formation of less compact regions within the spheroid. This structural alteration may facilitate the diffusion of therapeutic agents toward inner cell layers, as drug penetration in spheroids is strongly influenced by their three-dimensional architecture and cellular organization, suggesting that treatment effects may arise from architectural disruption rather than size reduction [27,40].

The increased structural complexity of spheroids has been associated with greater resistance to chemotherapeutic agents [31]. In line with this, TMZ (500 µM) exerted limited effects during the formation phase, with no detectable reduction in cell viability in GL15 and only a modest decrease of 12.15% in U343 spheroids, contrasting with the reductions observed under 2D conditions (19.02% in GL15 and 45.58% in U343). Notably, rutin exerted stage-dependent effects on spheroid development. During the formation phase, although TMZ (500 µM) alone showed limited efficacy, its combination with rutin (15 µM) markedly enhanced the cytotoxic effect, reducing cell viability by approximately 90.01% in GL15 and 63.89% in U343 spheroids. This effect was accompanied by modulation of cell aggregation from 48 h onward, indicating reduced cohesion and altered compaction. In contrast, established spheroids exhibited greater resistance to TMZ, with no detectable reduction in cell viability in GL15 and only a modest decrease of 7.37% in U343 following TMZ treatment (1000 µM). However, when combined with rutin (30 µM), TMZ significantly increased treatment efficacy, reducing cell viability by 94.80% in GL15 and 31.35% in U343. Under these conditions, rutin, alone or in combination with TMZ, increased spheroid size and entropy, even in the presence of cell death, indicating structural disorganization that may influence treatment response. In this context, spheroid models more closely recapitulate tumor tissue, exhibiting altered expression of genes related to proliferation, adhesion, and DNA repair, as well as increased resistance to chemotherapeutic agents compared to 2D cultures [31]. Additionally, the spatial organization of these models not only influences drug penetration and cellular responses but also reflects a resistance-dependent effect, in which more chemoresistant spheroids exhibit greater compaction while more sensitive ones display structural disorganization [41]. This differential adaptation to therapeutic stress may underlie the enhanced effect of rutin in combination with TMZ in resistant cells, consistent with studies showing that flavonoids modulate TMZ response by targeting survival mechanisms [42].

In addition, we investigated the impact of rutin on GBM cell migration. TMZ increased migration, particularly in resistant cells, consistent with evidence that resistance is associated with enhanced motility, cytoskeletal reorganization, including F-actin remodeling, and phenotypic adaptation to therapeutic stress [43]. These findings suggest that TMZ resistance involves not only cytoprotective mechanisms but also the acquisition of invasive traits. In contrast, rutin reduced migration and reversed the TMZ-induced pro-migratory effect after 48 h of exposure, as well as decreasing the migration radius. This effect was accompanied by increased expression of MMP2, laminin, and fibronectin. However, despite their established roles in ECM remodeling, this upregulation did not result in increased migration, indicating that ECM remodeling alone is insufficient to sustain cell motility. In addition to matrix degradation, MMPs regulate ECM architecture and signaling in a context-dependent manner [44], and uncoordinated remodeling may impair the structural conditions required for effective cell movement. However, invasion assays would be necessary to determine whether these molecular changes translate into functional invasive behavior. In GBM, the ECM functions as an active component of the tumor microenvironment, integrating inflammatory cues and intracellular pathways involved in tumor progression [45]. Signaling axes such as IL-6/STAT3 and PI3K/AKT regulate both ECM remodeling and migration [32,33]. Within this framework, our data show that increased fibronectin and laminin expression occurs alongside reduced migration and attenuation of inflammatory signaling. Consistently, previous studies report similar ECM upregulation following rutin exposure without a corresponding increase in migratory capacity [46]. Together, these findings suggest that ECM accumulation, when not coordinated with pro-migratory signaling, may impair rather than promote cell movement. Further investigation into cytoskeletal organization, focal adhesion dynamics, and integrin signaling is needed to clarify the mechanisms underlying this imbalance.

Indeed, evidence indicates that TMZ increases ROS levels, which act as early signals for the activation of pathways such as PI3K/Akt and IL-6/STAT3, contributing to cellular adaptation, tumor progression, and chemoresistance [14]. These processes are linked to redox-dependent signaling networks in GBM, in which oxidative imbalance modulates pathways that promote tumor survival and therapeutic resistance [47]. Consistently, our data show that TMZ increased ROS levels in both cell lines, corroborating the premise that ROS induction represents an early response to treatment. In parallel, TMZ also modulated inflammatory signaling, increasing IL-6 expression in U343 spheroids and differentially regulating STAT3 expression, with upregulation in GL15 and downregulation in U343, reinforcing the involvement of the IL-6/STAT3 axis in treatment-associated responses. In contrast, rutin reduced ROS levels even in the presence of TMZ and downregulated IL-6 and STAT3 expression, both alone and in combination with TMZ. This effect was accompanied by increased cytotoxicity, suggesting that disruption of redox-inflammatory signaling may impair adaptive survival mechanisms. In addition, rutin modulated components of the PI3K/Akt pathway, particularly under combined treatment, indicating coordinated interference with pro-survival signaling [18,48]. Although changes in STAT3 and Akt expression were observed, phosphorylation status (p-STAT3 and p-Akt), which more directly reflects pathway activation, was not assessed. Therefore, these results should be interpreted as modulation of pathway components rather than definitive evidence of pathway activation. Altogether, these findings indicate that redox and inflammatory signaling pathways are functionally integrated in GBM adaptive responses. By interfering with these axes, rutin appears to limit tumor cell survival, contributing to the increased sensitivity to TMZ observed in our experimental models.

In this context, rutin also modulated immunometabolic parameters associated with the IDO1–KP, a central axis often linked to tumor immune evasion through enhanced tryptophan degradation and kynurenine accumulation [34]. In stable spheroids, rutin treatment increased L-kynurenine levels, accompanied by a trend toward increased IDO1 expression, suggesting activation of this pathway. However, under the pretreatment (chemosensitization) condition, distinct effects were observed. Both TMZ and rutin alone reduced kynurenine levels compared to control, whereas the combination increased kynurenine levels relative to TMZ alone, indicating that the response depends on both the experimental context and the treatment sequence. These findings suggest that modulation of the IDO1–KP by rutin does not follow a linear pro-tumor pattern. Although this pathway is classically associated with immune evasion, its activation in our models occurred alongside reduced migration and increased cytotoxicity, indicating that immunometabolic alterations may be uncoupled from pro-survival phenotypes. Thus, our data support a context-dependent regulation, potentially influenced by the interplay between redox status, spheroid structural organization, and inflammatory signaling. Although IDO1 expression was assessed at the mRNA level and L-kynurenine levels were used as an indirect functional readout, IDO1 enzymatic activity was not directly measured, which represents a limitation of this study. Nonetheless, the concordance between these parameters supports the proposed interpretation.

Flavonoids have been investigated as modulators of chemoresistance in GBM [42]. In this context, our data show that pretreatment with rutin (5 μM) induces structural alterations in spheroids, characterized by increased entropy and reduced architectural homogeneity. These changes were accompanied by enhanced TMZ (500 μM)-induced cytotoxicity, reinforcing that rutin-mediated structural modulation facilitates drug sensitivity in three-dimensional models. Given the association between spheroid architecture and drug resistance, these findings corroborate the premise that disruption of structural organization contributes to improved therapeutic response. In addition, treatment sequence influenced parameters associated with cellular response. The reduction in NOS2 expression following rutin pretreatment, even in the presence of TMZ, indicates modulation of treatment-induced inflammatory signaling. Similarly, modulation of kynurenine levels under combined conditions, in contrast to single treatments, reinforces the context-dependent nature of this response [34]. Taken together, these findings indicate that rutin pretreatment enhances TMZ efficacy in a sequence-dependent manner, at least in part through modulation of spheroid structural organization. Future studies should assess whether structural modulation influences drug penetration and distribution within spheroids, to better define its impact on therapeutic response.

Taken together, our data demonstrate that rutin enhances TMZ responses by modulating redox balance and migratory behavior, while regulating immunometabolic pathways and contributing to the reprogramming of chemoresistance in GBM. These effects are accompanied by structural remodeling of tumor spheroids, highlighting three-dimensional architecture as a determinant of therapeutic response. Our findings support an integrated adaptive network involving ECM remodeling, redox-inflammatory signaling, and kynurenine metabolism, providing mechanistic insight into the tumor response. The influence of treatment sequence further underscores the dynamic nature of these processes. Although the mechanisms linking structural remodeling to drug response and pathway activation remain to be fully defined, and validation in more complex systems is warranted, further investigation is needed to determine whether the metabolic responses observed in primary astrocytes under 2D conditions are preserved in three-dimensional models. Although additional experiments such as formal drug interaction analysis, invasion assays, and phosphorylation studies would further strengthen the mechanistic interpretation, the current findings consistently demonstrate the biological effects of rutin across multiple complementary approaches, supporting the robustness of the observed responses. Our results support a model in which rutin reprograms key adaptive processes underlying chemoresistance, positioning it as a promising adjuvant strategy to enhance TMZ efficacy in GBM.

## 5. Conclusions

The flavonoid rutin enhances TMZ action in GBM by modulating adaptive resistance mechanisms, leading to reduced viability and migration, as well as structural reorganization of spheroids in 3D models. These effects are associated with coordinated regulation of redox balance, pro-survival signaling, and immunometabolic pathways, and are influenced by treatment sequence. Collectively, these findings support rutin as a promising adjuvant strategy to overcome chemoresistance in GBM.

## Data Availability

Data are contained within the article.

## Abbreviations

The following abbreviations are used in the manuscript:

2D	Two-dimensional
3D	Three-dimensional
ACTB	Beta-actin
AKT	Protein kinase B
AnaSP	ANAlyse SPheroids
ANOVA	Analysis of variance
BSA	Bovine serum albumin
cDNA	Complementary DNA
DAPI	4′,6-diamidino-2-phenylindole
DCF	2′,7′-dichlorofluorescein
DCFH-DA	2′,7′-dichlorodihydrofluorescein diacetate
DiOC	3,3′-dioctadecyloxacarbocyanine perchlorate
DiOC_18_(3)	3,3′-dioctadecyloxacarbocyanine perchlorate
DMEM	Dulbecco’s Modified Eagle Medium
DMSO	Dimethyl sulfoxide
ECL	Enhanced chemiluminescence
ECM	Extracellular matrix
EDTA	Ethylenediaminetetraacetic acid
EGTA	Ethylene glycol tetraacetic acid
FBS	Fetal bovine serum
GAPDH	Glyceraldehyde-3-phosphate dehydrogenase
GBM	Glioblastoma
hGBM	Human glioblastoma
HPRT1	Hypoxanthine phosphoribosyltransferase 1
HRP	Horseradish peroxidase
IC_50_	Half-maximal inhibitory concentration
IDO	Indoleamine 2,3-dioxygenase
IDO1	Indoleamine 2,3-dioxygenase 1
IL-6	Interleukin-6
KP	Kynurenine pathway
MMP2	Matrix metalloproteinase-2
MTT	3-(4,5-dimethylthiazol-2-yl)-2,5-diphenyltetrazolium bromide
NO	Nitric oxide
NOS	Nitric oxide synthase
NOS2	Nitric oxide synthase 2
PBS	Phosphate-buffered saline
PI	Propidium iodide
PI3K	Phosphoinositide 3-kinase
PVDF	Polyvinylidene difluoride
RNA	Ribonucleic acid
ROS	Reactive oxygen species
RT-qPCR	Quantitative real-time polymerase chain reaction
SDS-PAGE	Sodium dodecyl sulfate–polyacrylamide gel electrophoresis
SEM	Standard error of the mean
STAT3	Signal transducer and activator of transcription 3
TBS-T	Tris-buffered saline with Tween-20
TCA	Trichloroacetic acid
TMZ	Temozolomide
